# Determinants of facility readiness for integration of family planning with HIV testing and counseling services: evidence from the Tanzania service provision assessment survey, 2014–2015

**DOI:** 10.1186/s12913-017-2809-8

**Published:** 2017-12-22

**Authors:** Deogratius Bintabara, Keiko Nakamura, Kaoruko Seino

**Affiliations:** 1 Department of Global Health Entrepreneurship, Division of Public Health, Graduate School of Tokyo Medical and Dental University, 1-5-45 Yushima, Bunkyo-ku, Tokyo, 113-8519 Japan; 2 Department of Public Health, College of Health Sciences, The University of Dodoma, P.O Box 259, Dodoma, Tanzania

**Keywords:** Facility readiness, Integration, Family planning, HIV testing and counseling, Tanzania

## Abstract

**Background:**

Global policy reports, national frameworks, and programmatic tools and guidance emphasize the integration of family planning and HIV testing and counseling services to ensure universal access to reproductive health care and HIV prevention. However, the status of integration between these two services in Tanzanian health facilities is unclear. This study examined determinants of facility readiness for integration of family planning with HIV testing and counseling services in Tanzania.

**Methods:**

Data from the 2014–2015 Tanzania Service Provision Assessment Survey were analyzed. Facilities were considered ready for integration of family planning with HIV testing and counseling services if they scored ≥ 50% on both family planning and HIV testing and counseling service readiness indices as identified by the World Health Organization. All analyses were adjusted for clustering effects, and estimates were weighted to correct for non-responses and disproportionate sampling. Descriptive, bivariate, and multivariate logistic regression analyses were performed.

**Results:**

A total of 1188 health facilities were included in the study. Of all of the health facilities, 915 (77%) reported offering both family planning and HIV testing and counseling services, while only 536 (45%) were considered ready to integrate these two services. Significant determinants of facility readiness for integrating these two services were being government owned [AOR = 3.2; 95%CI, 1.9–5.6], having routine management meetings [AOR = 1.9; 95%CI, 1.1–3.3], availability of guidelines [AOR = 3.8; 95%CI, 2.4–5.8], in-service training of staff [AOR = 2.6; 95%CI, 1.3–5.2], and availability of laboratories for HIV testing [AOR = 17.1; 95%CI, 8.2–35.6].

**Conclusion:**

The proportion of facility readiness for the integration of family planning with HIV testing and counseling in Tanzania is unsatisfactory. The Ministry of Health should distribute and ensure constant availability of guidelines, availability of rapid diagnostic tests for HIV testing, and the provision of refresher training to health providers, as these were among the determinants of facility readiness.

**Electronic supplementary material:**

The online version of this article (10.1186/s12913-017-2809-8) contains supplementary material, which is available to authorized users.

## Background

The HIV pandemic remains a major public health problem worldwide, and its effects are especially devastating in Sub-Saharan Africa (SSA) [1]. Approximately, 25.5 million (69%) of the estimated 36.7 million people living with HIV globally in 2016 were from SSA, with 19.4 million living in East and Southern Africa, a region that accounted for about 44% of new HIV infections globally [2]. Moreover, adolescents and young adults, especially women aged 15–24 years old, were reported to be at high risk of acquiring new HIV infection [2, 3]. This burden of the HIV pandemic in SSA is in parallel with the challenges of the unmet needs for family planning (FP), which results in a high fertility rate [4]. Previous reports have shown that about 53 million women in this region want to prevent pregnancy but lack access to contraception [5, 6]. A high unmet need for FP in SSA has also been reported among HIV-positive women [7, 8], which has compromised efforts to prevent about 160,000 HIV-positive newborns annually due to unintended pregnancies [9–12].

Countries with the greatest HIV burdens also have high unmet needs for FP, and the same people that are at risk of unintended pregnancy are also at risk of HIV infection [13]. The incidence of unintended pregnancies remains alarmingly high among HIV-positive women in SSA [14], but the integration of FP into HIV programs has been underutilized [15, 16]. Demographic and Health Survey (DHS) data analysis indicated that about 14% of HIV-positive women in six African countries have unmet needs for FP, although they are in normal contact with the health system for HIV care [17].

In 2009, about 1.4 million people were estimated to be living with HIV in Tanzania; approximately 200,000 of these being children under 15 years of age, and 90% of them had acquired the infection through mother-to-child transmission (MTCT) [18], which is the second most common method of HIV transmission [19]. The use of FP is one of the comprehensive strategies available for the prevention of MTCT as well as unintended pregnancies among HIV-positive women or couples, however, this strategy is not useful in case HIV positive women or couples desire to have children [20]. Despite the advantages of FP for HIV-positive and non-HIV-positive women and couples, in Tanzania, about 68% of women of reproductive age are currently not using any modern method of contraception and 22% have unmet needs for FP [21]. This places Tanzania among the countries with a high fertility rate (5.2 births per woman) [21], which is slightly above the African average of 4.7 births per women reported in 2015 [22].

Prior to 2008, FP and HIV services were implemented under different and independent operational structures in Tanzania [13, 23]. These structures arose even though the unmet need for FP and the HIV epidemic share similar backgrounds, such as poverty, poor access to health services, and gender inequality [13]. Clients looking for FP services and those seeking HIV testing and counseling services (HTC) share several needs and concerns [24]. Therefore, providing these two services separately results in several unnecessary trips as well as adding to the financial burden, high level of stigma, and lack of follow-up visits [25]. On the other hand, integrating these services enables health facilities to offer FP services to those at risk of HIV and those living with HIV [11, 25, 26]. This reduces the spread of HIV infection by maximizing the use of contraception among these groups [12, 27]. Several studies have provided evidence confirming the feasibility and effectiveness of integrating these services [28–33]. In addition, several global policy reports, national frameworks, and programmatic tools and guidance suggest that providers should integrate FP into HTC services to achieve universal access to reproductive health care and HIV prevention [34–36].

Facility Readiness for the Integration of FP with HTC services (FRIFHS) reflects the willingness or ability of a facility, regardless of its level, to offer FP and HTC services at a single time point to ensure and maximize collective health outcomes [13, 37, 38]. Health facilities in Tanzania are categorized into three groups depending on the population coverage, expertise, and availability of services: dispensaries/clinics, which are the lowest level of health facilities that provide preventative and curative outpatient services, including normal delivery and are expected to serve up to a total of 10,000 inhabitants in local communities; health centers, which offer all services available in the dispensaries but have further capacity to provide inpatient services and are also expected to perform some surgical interventions, e.g., emergency obstetric care; and hospitals, which provide similar services to health centers but at a greater level of expertise given the higher levels of clinical and nursing care capability and laboratory/radiology diagnostic capacity. Despite the differences in functions, all three levels are expected to provide primary health care, such as HTC and FP services [39].

In 2008, the government of Tanzania established a policy supporting the integration of FP with HTC services. Therefore, the national policies for HIV/AIDS care and treatment were revised to include FP as a core component [23]. The FP components were included in at least one of the six major HIV policies and guidelines, while “HIV/AIDS” components were included in at least one of the four major FP and Reproductive Health (RH) policies and guideline documents [40]. Despite this effort to establish integration policy and the highlighted benefits of integrating FP with HTC services [41–43], only 38% of HIV-infected women attending care and treatment clinics (CTC) in Tanzania reported having discussed FP with care providers. Around 70% of those who missed the discussion did not intend to become pregnant within the following 6 months [23]. This may be due to poor implementation of integration strategies due to unknown factors. However, some studies have attempted to assess client factors associated with the use of integrated services [44–47]. Possible issues that may compromise integrated services have been proposed, such as procurement problems, the lack of continuous supply of equipment, and expenses associated with laboratory tests [48–50]. Furthermore, inadequate training, lack of supervision, heavy workload, lack of incentive, and staff burnout have been reported as provider-related barriers to the provision of integrated services [50–55].

Despite these factors, the majority of studies have concentrated on the availability of services [48–50, 56, 57] and have not examined whether the facilities are actually ready to offer the desired integrated services. In fact, the availability of services does not guarantee that clients will receive integrated services if the facilities or providers are not willing to offer these services [58, 59]. For example, integration of FP with HIV services was introduced in Tanzania in 2008, but the majority of clients are still unable to receive integrated care [9]. Therefore, facility readiness is an important area of study, as little is actually known regarding this issue. The present study was performed to assess the current extent and determinants of FRIFHS in Tanzania using a nationwide representative sample.

## Methods

### Data source

This study used data from the Tanzania Service Provision Assessment (Tanzania SPA) Survey, conducted between 20 October 2014 and 21 February 2015. The survey was undertaken by Tanzania’s National Bureau of Statistics (NBS) and the Office of the Chief Government Statistician (OCGS) in collaboration with the Ministry of Health and Social Welfare (MoHSW) – Mainland, and the Ministry of Health (MoH) – Zanzibar. This survey is the second facility-based survey conducted in Tanzania, following the one conducted in 2006. The survey, funded by the United States Agency for International Development (USAID) [60], was designed to provide information on the availability of basic and essential health care services and on service readiness. It assessed the presence and functioning of components essential for quality service delivery for child health, maternal and newborn care, FP, antenatal care, sexually transmitted infections, HIV/AIDS, and non-communicable diseases [60].

### Sample size and sampling procedure

The methodology of this survey is reported elsewhere, but briefly, from a list of 7102 verified (active) health facilities as a sampling frame; 1200 facilities were randomly selected for inclusion in the survey. The sample was designed to provide nationally representative results according to facility type and managing authority, and regionally representative results for all 25 Tanzania mainland regions and five Zanzibar regions [60]. However, of the 1200 health facilities sampled, seven refused to participate in the survey, four had closed down, and one facility could not be reached. Finally, a total of 1188 facilities took part in this survey.

### Data collection

The 2014–2015 Tanzania SPA survey data were collected between 20 October 2014 and 21 February 2015, and some facilities that were not covered previously were revisited from 2 to 13 March 2015. The survey used four main types of data collection tools: Facility Inventory questionnaire; Health Provider Interview questionnaire; Observation Protocols for antenatal care (ANC), FP, and services for sick children; and Exit Interview questionnaires for ANC and FP clients and for caregivers of sick children whose consultations were observed [60].

### Data processing and management

An online DHS website request for permission to access the Tanzania SPA survey dataset was performed by the first author on 29 October 2016. Two days later, the authors received full online access to all four (Facility, Health Provider, Observation, and Client Exit) files of Tanzania SPA survey dataset. Most of the data analyzed in this study were obtained from the Facility Inventory file, but one variable regarding staff that had received refresher training was obtained from the Health Provider file. The data in these file were edited, cleaned, and recoding was performed for variables of interest to obtain meaningful information for addressing our research questions. Data cleaning was performed by calculating the frequencies and sorting. Finally, these two files were merged in Stata together into new file (dataset) using facility identification as a unique identifier of these two files. As the Facility Inventory was used as the master file and Provider was used as the using file, the *merge* command in Stata was performed at 1:1; therefore, the unit of analysis of the new file remained at the facility level. After merging, 12 cases from the Facility Inventory file were not matched. This was because 12 facilities sampled were not interviewed. Therefore, they were regarded as missing variables and were excluded from further analysis. As the facilities sampled were not evenly distributed and the response rate may be very different between regions or facility type, over- and under-sampling were performed in the regions with fewer and more facilities, respectively, prior to data collection. Before analysis, therefore, facility weight was applied to restore the actual representativeness and corrected for sampled data.

### Operational definitions

**Facility readiness:** defined in this study as the willingness or state of the health facility to provide FP and HTC as integrated services.

**Refresher training:** defined as on-the-job training provided to health providers without taking into consideration their roles to update their knowledge, skills, and technical competence to improve health care related to the services offered to clients.

### Measurement of variables

#### Dependent variable

The FRIFHS was measured based on the scores of FP and HTC service readiness indices. The scores were determined using the WHO approach, and FP and HTC readiness indicators were identified according to the WHO Service Availability and Readiness Assessment (SARA) Manual [38]. Using this approach, the FP service readiness index was categorized into three domains; staff and guidelines, equipment and medicines/commodities. The first domain consisted of staff and guidelines, which had two indicators, i.e., guidelines on FP and staff trained in FP. The facilities with guidelines and at least one staff member that had received refresher training in FP within 24 months were categorized as “Yes,” while those without such guidelines or at least one staff member receiving refresher training in FP within 24 months were categorized as “No.” The second domain was equipment, which had one indicator, i.e., the presence of blood pressure (BP) apparatus. Facilities with BP apparatus were categorized as “Yes,” while those without were categorized as “No.” The third domain was medicine and commodities, which had four indicators, i.e., the availabilities of progestin-only pills, combined oral pills, injectable contraceptives (combined or progestin-only), and condoms. Facilities with the availability of these means of contraception were categorized as “Yes,” while those without were categorized as “No.” The HTC service readiness index was categorized into three domains. The first domain was staff and guidelines, which had two indicators, i.e., guidelines and staff trained in HTC. Facilities with guidelines and at least one staff member that had received refresher training in HTC within 24 months were categorized as “Yes,” while those without such guidelines and no staff members receiving HTC refresher training within 24 months were categorized as “No.” The second domain was laboratory capacity. Facilities with the capability to perform rapid diagnostic tests or ELISA for HIV were categorized as “Yes,” while those without were categorized as “No.” The third domain was supplies/commodities, which had one indicator; facilities with the availability of condoms at testing and counseling sites were categorized as “Yes,” while those without were categorized as “No.”

Each index (FP and HTC service readiness) was then totaled by adding the presence of each indicator, with equal weight given to each of the domains and each of the indicators within the domains. As the target was 100%, each domain accounted for 33.3% (100%/3) of the index. The percentage for each indicator within the domain was equal to 33.3% divided by the number of indicators in that domain. The FP and HTC service readiness indices for each facility were then calculated by summing the percentages. Facilities with scores of at least 50% for the FP service readiness index and 50% for the HTC service readiness index were considered to be ready for the integration of FP with HTC services (i.e., FRIFHS), while those that scored less than 50% for each index were not considered to be ready (i.e., not FRIFHS) [38, 61]. An additional table in word document file summarizes this measurement procedure in more details [see Additional file 1].

#### Independent variables

Facility type was coded as “1” for a hospital, “2” for a health center, and “3” for a clinic or dispensary. The residence was coded as “0” for urban and “1” for rural. Managing authority was coded as “0” for a private facility and “1” for the public facility. Routine management meetings were coded as “Yes” for facilities reporting that they performed routine management meetings at least on a quarterly basis and “No” for those that reported having no such meetings at least on the quarterly basis. External supervision was coded as “Yes” for facilities reporting having receiving external supervision in the past 3 months and “No” for those without such supervision. Location was categorized as “0” for Zanzibar and “1” for the mainland. FP and HTC guidelines were categorized as “0” for facilities without guidelines for both FP and HTC and “1” for facilities with guidelines for both FP and HTC. Staff trained in FP and HTC were categorized as “0” for facilities without staff trained in both FP and HTC and “1” for facilities with staff trained in both FP and HTC. Laboratory capacity was categorized as “0” or “1” for facilities without and with laboratory capacity to test for HIV, respectively.

### Statistical analysis

Data were analyzed using Stata 14 (StataCorp, College Station, TX). For all analyses, the “svy” set command in Stata was used to adjust for the complex sampling design used by Tanzania SPA survey. All estimates were weighted to correct for non- responses and disproportionate sampling.

In descriptive analyses, categorical variables were summarized using proportions and then presented in tables and graphs. An unadjusted logistic regression model was fitted to examine whether there were any associations between the dependent variable (FRIFHS) and the independent (health facility) variables (location, residence, facility type, managing authorities, management meetings, FP and HTC guidelines, staff trained in FP and HTC, laboratory capacity for HIV testing, and external supervision) using multilevel modeling. All variables with *P* < 0.2 were fitted into the multiple logistic regression models using the stepwise (backward elimination) method to test for the association of each variable with the dependent variable using a 5% significance level. The resulting final model included all of the variables that determined the FRIFHS. The *P*-value and 95% confidence interval (CI) for the odds ratio (OR) were used to confirm the significance of the associations. In all analyses, *P* < 0.05 was taken to indicate statistical significance.

### Ethical considerations

The 2014–2015 Tanzania SPA survey was approved by Tanzania’s National Institute for Medical Research (NIMR), the Zanzibar Medical Ethics and Research Committee (ZAMREC), and the Institutional Review Board of ICF International in the USA. Before interviews were performed, informed consent was obtained from the manager, the person-in-charge of the facility, or the most senior health worker responsible for client services present at the facility. The respondents were adequately informed regarding all relevant aspects of the study, including its aim and interview procedures. Respondents that accepted for their facilities to participate in the study signed informed consent.

## Results

### Background characteristics of selected health facilities

Table 1 presents a summary of the background characteristics of included health facilities. A total of 1188 health facilities representing a 99% response rate were included in the analysis. The majority (1044, 95.3%) were from the Tanzania mainland, and 864 (72.7%) were located in rural settings. Most of the facilities (1013, 85.3%) were dispensaries or clinics, and 857 (72.2%) were public facilities. The majority (922, 77.6%) reported having routine management meetings. Among all of the facilities, 523 (44.1%) reported having both FP and HTC guidelines in place, but only 136 (11.5%) reported having at least one health provider that had received training in both FP and HTC services. The majority (842, 70.9%) reported having a laboratory for HIV testing, but only a few facilities (34, 3.0%) reported receiving external supervision from a higher ranking facility (Table 1).

**Table 1:** Percentage distribution of surveyed facilities according to background characteristics, Tanzania SPA 2014–15 (*n* = 1188)

Variable	Number (weighted) (*n*)	Percentage (%)
Facility location		
Zanzibar	44	3.7
Mainland	1144	96.3
Managing authority		
Public	857	72.2
Private	331	27.8
Facility type		
Hospital	46	3.9
Health center	129	10.8
Clinic & dispensary	1013	85.3
Facility residence		
Urban	324	27.3
Rural	864	72.7
Routine management meetings		
No	266	22.4
Yes	922	77.6
FP and HTC guidelines		
Not available	664	55.9
Available	524	44.1
Staff trained for FP and HTC		
Not available	1052	88.5
Available	136	11.5
Laboratories for HIV testing		
Not available	346	29.1
Available	842	70.9
External supervision		
Not done	34	3.0
Done	1154	97.0

### Availability of services and health facility readiness for offering FP and HTC services

Table 2 shows the distributions of availability and readiness of facility for offering FP and HTC services. The majority of the health facilities included in the study reported the availability of HTC (1039, 87.5%) and availability of FP services (948, 79.8%), while (915, 77.0%) reported offering both services. Less than half of the interviewed health facilities (487, 41.0%) reported having both FP guidelines and at least one health provider that had received refresher training in FP services, while 677 (57.0%) reported having BP apparatus as important equipment to provide FP services, but only 307 (25.9%) health facilities reported having progestin-only pills, combined oral pills, injectable contraceptives (combined or progestin-only), and condoms. Furthermore, about one third (380, 32.0%) of the health facilities reported having both HTC guidelines and at least one health provider that had received refresher training in HTC services. However, the majority (842, 70.9%) reported having a laboratory capacity for HIV testing and 674 (56.7%) reported the availability of condoms at the testing and counseling site.

**Table 2:** Percentage distribution of health facilities with the availability and readiness to offer FP and HTC services, Tanzania SPA 2014–15 (*n* = 1188)

Variable	*Number (weighted) (*n*)	*Percentage (%)
Service availability		
FP	948	79.8
HTC	1039	87.5
Both	915	77.0
Components of FP readiness index		
Staff and guidelines	487	41.0
Equipment	677	57.0
Medicine/commodities	307	25.9
Components of HTC readiness index		
Staff and guidelines	380	32.0
Laboratory capacity to test HIV	842	70.9
Supplies/commodities	674	56.7
Overall facility readiness		
FP	720	60.6
HTC	701	59.0
Integrated services	536	45.1

Based on the WHO readiness indicator score, only 720 (60.6%) and 701 (59.0%) facilities were ready to offer FP and HTC services, respectively, while only 536 (45.0%) were ready to offer integrated services.

### Determinants of facility readiness for integration of FP with HTC services

Table 3 presents the results of bivariate and multivariate analyses. In bivariate analysis, facility location, type of managing authority, facility residence, routine management meetings, availability of both FP and HTC guidelines, availability of staff trained in both FP and HTC, and availability of a laboratory with capacity for HIV testing were found to be significantly associated with FRIFHS.

**Table 3:** Determinants of FRIFHS in bivariate and multivariate analyses, Tanzania SPA 2014–2015 (*n* = 1188)

Variable	COR [95%CI]	*P*-value	AOR [95%CI]	*P*-value
Facility location				
Zanzibar	1		1	
Mainland	1.6 [1.1–2.5]	0.043	1.3 [0.6–2.7]	0.871
Managing authority				
Private	1		1	
Public	8.5 [5.2–13.8]	0.000	3.2 [1.9–5.6]	0.000
Facility type				
Hospital	1			
Health center	1.1 [0.8–1.7]	0.478	–	
Clinic & dispensary	1.2 [0.8–1.7]	0.423		
Facility Residence				
Urban	1		1	
Rural	2.6 [1.7–3.9]	0.000	1.1 [0.6–1.9]	0.759
Routine management meetings				
No	1		1	
Yes	1.7 [1.1–2.6]	0.010	1.9 [1.1–3.3]	0.014
External supervision				
Not done	1		1	
Done	3.7 [0.8–16.9]	0.093	1.6 [0.2–11.2]	0.656
FP and HTC guidelines				
Not available	1		1	
Available	7.1 [4.8–10.2]	0.000	3.8 [2.4–5.8]	0.000
Staff trained in FP and HTC				
Not available	1		1	
Available	4.7 [2.5–8.7]	0.000	2.6 [1.3–5.2]	0.006
Laboratories for HIV testing				
Not available	1		1	
Available	32.8 [16.5–65.1]	0.000	17.1 [8.2–35.6]	0.000

“1” indicates the categories considered as references when comparing odds ratios

--- Variable was not included in multivariate analysis because *P* > 0.2 in bivariate analysis

In multiple logistic regression analysis, the baseline model included the following variables: FRIFHS as a dependent variable and facility location, managing authority, facility residence, routine management meetings, external supervision, FP and HTC guidelines, staff trained in FP and HTC, and laboratory capacity for HIV testing as independent variables. In the final model, the likelihood of FRIFHS was three times higher among public health facilities (owned by the government) than privately owned facilities [AOR = 3.2; 95%CI, 1.9–5.6]. Similarly, the likelihood of FRIFHS among facilities that reported having routine management meetings was double that in facilities without such meetings [AOR = 1.9; 95%CI, 1.1–3.3]. In addition, the likelihood of FRIFHS was fourfold higher among facilities that reported having both guidelines for FP and HTC services than in those without both or either of these guidelines [AOR = 3.8; 95%CI, 2.4–5.8]. Furthermore, the likelihood of FRIFHS was 2.5 times higher among facilities that reported having at least one staff member that had received training for both FP and HTC services than in those without such staff training [AOR = 2.6; 95% CI, 1.3–5.2]. Finally, the likelihood of FRIFHS was 17 times higher for facilities with, than without a laboratory capable of performing HIV testing [AOR = 17.1; 95% CI, 8.2–35.6].

## Discussion

This study aimed to assess the extent and determinants of FRIFHS in Tanzania. To our knowledge, this is the first study to assess the readiness for integration of FP with HTC services in this region using recommended WHO service readiness indicators, and data obtained from a nationally representative sample with “health facility” as the sampling unit. The results presented here indicated an unsatisfactory proportion of FRIFHS in Tanzania. However, the majority of these facilities showed the increased capability of offering these services independently compared to 10 years ago [40, 62]. In addition, the present study indicated that managing authorities, routine management meetings, the availability of FP and HTC guidelines, refresher staff training in FP and HTC, and the availability of laboratories with capacity for HIV testing were significant determinants of FRIFHS.

The low proportion of FRIFHS observed in this study was in contrast to the findings of a study performed in Uganda to assess the integration of routine rapid HIV screening in urban FP clinics, which indicate that about 78% of staff were ready for integration and rated it as a successful effort to expand routine HIV testing in FP clinics [63]. This difference in findings may have been because our study involved both urban and rural facilities and also included all types of health facilities, whereas the study performed in Uganda included only urban facilities. In addition, the low proportion of FRIFHS observed in the present study may have been because the integration of FP with HTC was treated as a composite variable obtained by considering several indicators unlike the method used in the previous study in which the readiness for integration was based on the willingness of the staff to integrate these two services.

In most countries, including Tanzania, FP and HTC services are provided through public and private health facilities even though private facilities are operated for profit [64, 65]. The results of the present study indicated that public health facilities were three times more likely to have integrated FP with HTC services than those in the private sector. Thus, public facilities are more likely to offer integrated services. This conclusion agreed with previous studies indicating the better provision of health services at public than at private facilities [66–69]. This finding may be because most public facilities do not have the mandate to choose the type of service to offer rather than to comply with government policies compared to private facilities where the choice of services offered is dependent on the capacity of investment and demand for services. In addition, as public facilities offer FP and HTC for free, while private facilities tend to charge for these services, this may reduce demand in the private sector and make it seem to not be a worthwhile investment. Public facilities receive allocated funds from the government budget, which in turn receives funds from development partners, resources from non-governmental organizations, out-of-pocket payments, and funds from health insurance, while private facilities depend on out-of-pocket payments only and some rely on health insurance [70, 71]. However, this finding was in contrast to those of previous studies performed in Pakistan [72], Jordan [73], Thailand [74], and Switzerland [75], and may have been due to the growth of health privatization policies in the latter countries. Privatization of the health sector is still new in Tanzania compared with the other countries mentioned above. Therefore, the majority of private health facilities still choose which types of services to offer for the purpose of profit.

Having regular meetings between the managers and staff members of a facility increases the effectiveness of providing appropriate services to clients. These meetings are sometimes used to monitor if the health workers are doing their job. The results of the present study indicated that the likelihood of FRIFHS among facilities that conducted routine management meetings was double that in those that did not. This difference may reflect that such meetings represent not only a form of monitoring that staff are performing their duties according to the appropriate standards but could also be a method to provide managers and staff members with the opportunity of discussing challenges hindering the provision of integrated services and allow them to propose and work on suitable solutions. These observations were in agreement with those of a previous study conducted in Kenya [59].

The availability of guidelines and staff training are essential to provide guidance, up-to-date knowledge and skills required to deliver integrated services. This study indicated that the proportion of health facilities with both guidelines (FP and HTC) and at least one health provider that had received training (for FP and HTC) was low in Tanzania. The study also indicated that facilities with both FP and HTC guidelines were four times more likely to have FRIFHS status than their counterparts. Thus, the presence of guidelines can improve health care decisions by providing clear guidance regarding what should be integrated [50, 76], but also encourages providers to adhere to standards of care [77]. Therefore, facilities with guidelines are more likely to integrate services. Similar findings have been reported in studies conducted in low- and middle-income countries [78].

In Tanzania, it is unusual to have high cadre health professionals at lower-level health facility hence an opportunity for other cadres to gain new skills and knowledge. Therefore, on-the-job refresher training provides all cadres of healthcare providers with the opportunity to acquire extra knowledge, skills, and technical competence regarding health services provided in their workplace. Furthermore, the provision of several services at a single point of contact requires healthcare providers to be sufficiently trained in all aspects of the services concerned to allow them to offer the desired services in an integrated form [51, 78]. The results of the present study indicated that the proportion of health facilities with at least one staff member trained in providing both FP and HTC was low in Tanzania. These facilities with at least one staff member with FP and HTC training were 2.5-fold more likely to have FRIFHS compared with their counterparts. This difference may have been because staff members that have received training for the services offered are more likely to practice what they have recently learned. Therefore, they are more likely to request the necessary equipment and medications as well as guidelines for their working station, thereby improving the overall integration of health services within their facilities.

The availability of equipment, medical supplies, and drugs were found to be major factors in the adoption of integrated HIV services [79–81]. The majority of health facilities in this study were found to have laboratories with the capacity for HIV testing (rapid diagnostic kits and ELISA), and these facilities were found to be more likely to have FRIFHS than their counterparts. This can be explained by the fact that the availability of rapid diagnostic kits and ELISA for HIV testing within a facility’s laboratory could be because of high HIV prevalence and increased awareness that creating high demand for the services hence these units tend to have them as the part of routine care provision. Our findings were comparable to those of a study performed in Lesotho [82].

The present study is the first to use the Tanzania SPA 2014–2015 dataset, which is a nationwide, representative sample of health facilities, with a response rate of 99%, suggesting that the present findings accurately reflect the present conditions in Tanzania. To improve our analysis of the determinants of FRIFHS, we also adjusted for clustering effects and weighted the analysis to correct for non-responses and disproportionate sampling. However, the present study also had some limitations due to its cross-sectional nature preventing causality assumptions, and therefore the results should be interpreted with caution. In addition, the lack of previous surveys to assess the status and determinants of facility readiness for integration of FP and HTC services using WHO service readiness indicators make it difficult to compare the present findings with those of other studies.

## Conclusions

Analysis of the Tanzania SPA 2014–2015 dataset in this study indicated that the proportion of FRIFHS is unsatisfactory, even though the majority of facilities were ready to offer these services separately. Significant determinants for FRIFHS were being a public facility, having routine management meetings, the presence of HTC and FP guidelines, regular refresher staff training about HTC and FP, and laboratories with the capacity to perform HIV testing.

For Tanzanian health facilities to integrate FP with HTC services, the government, acting through MoHCDGEC, should distribute and ensure constant availability of FP and HTC guidelines and rapid diagnostic tests for HIV, in addition to providing FP and HTC in-service training to health providers. In addition, the MoHCDGEC should make regular visits and provide incentives to private health facilities to encourage the provision and integration of FP with HTC services.

## Additional file

Additional file 1: Summary of measurement procedure of the outcome variable FRIFHS. Table summarizing measurement procedure of the outcome variable FRIFHS. (DOCX 18 kb)

## Abbreviations

AIDS: Acquired immunodeficiency syndrome
AOR: Adjusted odds ratio
COR: Crude odds ratio
ELISA: Enzyme-linked immunosorbent assay
FP: Family planning
FRIFHS: Facility Readiness for the Integration of FP with HTC Services
HIV: Human immunodeficiency virus
HTC: HIV testing and counseling
MoHCDGEC: Ministry of Health, Community Development, Gender, Elderly, and Children
MTCT: Mother-to-Child Transmission
SPA: Service provision assessment
SSA: Sub-Saharan Africa
WHO: World Health Organization
