# Supplementary material for: Determinants of facility readiness for integration of family planning with HIV testing and counseling services: evidence from the Tanzania service provision assessment survey, 2014–2015
Source: BMC Health Serv Res. 2017 Dec 22;17:844. doi: 10.1186/s12913-017-2809-8 (PMC5741912; doi:10.1186/s12913-017-2809-8)
Supplement: Additional file 1: — Summary of measurement procedure of the outcome variable FRIFHS. Table summarizing measurement procedure of the outcome variable FRIFHS. (DOCX 18 kb) [file 12913_2017_2809_MOESM1_ESM.docx]

**Additional file 1 Summary of measurement procedure of outcome variable FRIFHS**

| **Domain** | **indicators** | **Measurement** | **Percent score (%)** | |
| --- | --- | --- | --- | --- |
|  |  |  | **Indicator** | **Domain** |
| **FP service readiness index** |  |  |  |  |
| Staff and guidelines | Guidelines for FP | Yes  No | 16.65  0.00 | 33.33 |
|  | At least one staff trained in FP | Yes  No | 16.65  0.00 |  |
| Equipment | BP apparatus | Yes  No | 33.33  0.00 | 33.33 |
| Medicines and commodities | Progestin-only | Yes  No | 8.33  0.00 | 33.33 |
|  | Combined oral pills | Yes  No | 8.33  0.00 |  |
|  | Injectable contraceptives | Yes  No | 8.33  0.00 |  |
|  | Availability of Condoms | Yes  No | 8.33  0.00 |  |
| Total FP readiness index score |  |  |  | 100.00 |
| **HTC service readiness index** |  |  |  |  |
| Staff and guidelines | Guidelines for HTC | Yes  No | 16.65  0.00 | 33.33 |
|  | At least one staff trained in HTC | Yes  No | 16.65  0.00 |  |
| Laboratory capacity | Availability of HIV rapid kits or ELISA test | Yes  No | 33.33  0.00 | 33.33 |
| Supplies and commodities | Availability of condoms | Yes  No | 33.33  0.00 | 33.33 |
| Total HTC readiness index score |  |  |  | 100.00 |
